# Platinum (IV) Recovery from Waste Solutions by Adsorption onto Dibenzo-30-crown-10 Ether Immobilized on Amberlite XAD7 Resin–Factorial Design Analysis

**DOI:** 10.3390/molecules25163692

**Published:** 2020-08-13

**Authors:** Oana Buriac, Mihaela Ciopec, Narcis Duţeanu, Adina Negrea, Petru Negrea, Ioan Grozav

**Affiliations:** 1 Faculty of Industrial Chemistry and Environmental Engineering, Politehnica University of Timişoara, 2 Piata Victoriei, RO 300006 Timisoara, Romania; oana.grad@upt.ro (O.B.); narcis.duteanu@upt.ro (N.D.); petru.negrea@upt.ro (P.N.); 2 Research Institute for Renewable Energies, Politehnica University of Timişoara, 138 Gavril Musicescu, RO 300501 Timisoara, Romania; ioan.grozav@upt.ro

**Keywords:** platinum (IV), recovery, dibenzo-30-crown-10, Amberlite XAD7 resin, adsorption, factorial design

## Abstract

Platinum is a precious metal with many applications, such as: catalytic converters, laboratory equipment, electrical contacts and electrodes, digital thermometers, dentistry, and jewellery. Due to its broad usage, it is essential to recover it from waste solutions resulted out of different technological processes in which it is used. Over the years, several recovery techniques were developed, adsorption being one of the simplest, effective and economical method used for platinum recovery. In the present paper a new adsorbent material (XAD7-DB30C10) for Pt (IV) recovery was used. Produced adsorbent material was characterized by X-ray dispersion (EDX), scanning electron microscopy (SEM) analysis, Fourier Transform Infrared Spectroscopy and Brunauer-Emmett-Teller (BET) surface area analysis. Adsorption isotherms, kinetic models, thermodynamic parameters and adsorption mechanism are presented in this paper. Experimental data were fitted using three non-linear adsorption isotherms: Langmuir, Freundlich and Sips, being better fitted by Sips adsorption isotherm. Obtained kinetic data were correlated well with the pseudo-second-order kinetic model, indicating that the chemical sorption was the rate-limiting step. Thermodynamic parameters (Δ*G*°, Δ*H*°, Δ*S*°) showed that the adsorption process was endothermic and spontaneous. After adsorption, metallic platinum was recovered from the exhausted adsorbent material by thermal treatment. Adsorption process optimisation by design of experiments was also performed, using as input obtained experimental data, and taking into account that initial platinum concentration and contact time have a significant effect on the adsorption capacity. From the optimisation process, it has been found that the maximum adsorption capacity is obtained at the maximum variation domains of the factors. By optimizing the process, a maximum adsorption capacity of 15.03 mg g^−1^ was achieved at a contact time of 190 min, initial concentration of 141.06 mg L^−1^ and the temperature of 45 °C.

## 1. Introduction

Platinum is widely used in energy production, electronic, health, jewellery and chemical industries, and due to its high chemical stability as well as high conductivity, its consumption has been experiencing a sharp increase during the past decades [1,2,3,4,5].

Over recent years, and due to natural resource scarcity and the growing demand for precious metals, there has been strong economic motivation for the recovery of precious metals from different industrial waste [6,7,8]. Platinum recovery is possible by using conventional methods such as: oxidation, precipitation, reduction, ion-exchange, liquid-liquid extraction, membrane filtration and adsorption [9,10,11,12,13,14]. A popular method used for recovery and recycling of metals is adsorption due to its low cost, easy operation, and simple system maintenance.

Over recent years, for selective recovery and separation of some noble metals was impetuously required to develop advanced materials with specific properties able to be used in adsorption process. Materials adsorbent properties were improved by chemical modification of the inorganic and organic solid supports, through impregnation with different extractants. Some of the materials with good adsorbent properties used for platinum recovery are: silica gels, clays [15,16], chitosan, chemically modified oxides by functionalization [17] and bio-sorbents (orange peel, grape wastes and rice husk) [18,19,20,21].

Statistics and statistical methods play an important role in planning, conducting, analysing and interpreting data obtained from experimental determinations. When more variables influence a particular feature of a process, the best strategy is to design an experiment so that it can draw reliable and cost-effective conclusions at the end of the process [22,23,24,25,26].

This research paper presents an original method used to prepare new adsorbent material with good efficiency for platinum recovery from wastewater and waste solutions. Used adsorbent was produced by functionalization of Amberlite XAD7 resin with dibenzo-30-crown-10-ether. This ether was chosen as extractant because of its well-known ability of crown ethers to bind metallic ions, whether they are symmetrically or unsymmetrically substituted.

Factorial design goal was to determine parameters and parameter settings value that have a significant influence on the adsorption process (contact time, initial adsorbent dose and temperature), to determine the process results to the desired values.

Thus, in the present paper, and in order to control the adsorption process used for platinum recovery from waste solutions, an experimental study was conducted by planning a Factorial Design (linear) and a Surface Response Design (nonlinear) experiment to improve the process and reduce overall costs.

## 2. Materials and Methods

### 2.1. Chemicals and Apparatus

Adsorbent material was obtained starting from Amberlite XAD7 resin (particle size 0.5–0.7 mm, surface area 380 m^2^ g^−1^), dibenzo-30-crown-10 ether (DB30C10), and nitrobenzene purchased from Merck Germany. In order to get the crown ether immobilized onto the solid support surface, the system was dried at 323 K for 24 h.

Platinum solution used during adsorption experiments was obtained from platinum chloride (Merck, Damstadt, Germany). Working standards for Pt (IV) were prepared by progressive dilution of standard Pt (IV) solutions with deionized (DI) water.

New prepared adsorbent material was characterized by using: Energy dispersive X-ray analysis and scanning electron microscopy (tests were performed using a FEG Quanta 250 scanning electron microscope—Thermo Fisher Scientific, Waltham, MA, USA), Fourier Transform InfraRed spectroscopy (spectra were recorded using a Bruker Platinum ATR-QL Diamond spectrometer—Bruker, Billerica, MA, USA), Brunauer, Emmett, Teller specific surface area (was determined by using a Quantachrome Nova 1200 E system). For all experimental determination the pH was measured by using a Mettler Toledo SevenCompact S210 pH meter. Zero point charge of XAD7-DB30C10 adsorbent material has been determined by keeping in contact 0.1 g of adsorbent material with KCl solutions with a concentration of 0.01 M, and pH between 2 and 13, for 60 min. After filtration has been measured the pH of the obtained solutions.

Pt (IV) concentration was measured by using a Varian SpectrAA 280 Fast Sequential Atomic Adsorption Spectrometer (Varian Inc, Melbourne, Australia) with air-acetylene flame at wavelength of 265.9 nm.

For batch experiments, a mechanical shaker bath Julabo SW23 (Julabo, Seelbach, Germany) with thermostatic and shaking control has been used.

### 2.2. Immobilization of the DB30C10 onto XAD7 Resin

**Figure 1:**
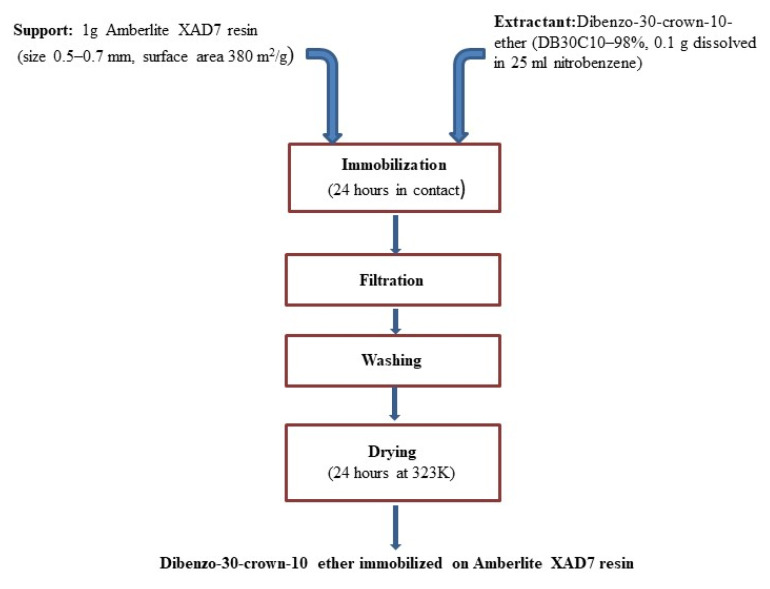
Preparation of XAD7-DB30C10 adsorbent material using Solid Impregnated Resin (SIR) method.

Used adsorbent material was produced by immobilization of DB30C10 ether on Amberlite XAD7 resin, by using Solid Impregnated Resin (SIR) dry method [27,28]. Thus, for preparation of this new material 0.1 g of DB30C10 ether were dissolved in 25 mL nitrobenzene, resulted solution was mixed with 1 g of Amberlite XAD7 resin. Obtained mixture was kept in contact for minimum 24 h, and after it was filtered, washed with DI water and dried at 323 K for 24 h. Schematically, the steps followed for preparation of the new adsorbent material used for Pt (IV) recovery are presented in Figure 1.

### 2.3. Influence of Physicochemical Parameters on Pt (IV) Adsorption

In present work was studied the influence of different physicochemical parameters (pH, contact time, temperature and initial concentration of platinum ions) for the adsorption process of Pt (IV) on XAD7-DB30C10 produced adsorbent.

pH influence on the Pt (IV) adsorption onto the XAD7-DB30C10 was studied by mixing 0.1 g of adsorbent with 25 mL of Pt (IV) solution, containing 25 mg Pt (IV) per L, having different pH values (2, 3, 4, 5, 6, 8, 9 and 10). In order to obtain the desired pH value, the solution pH was adjusted by adding HNO_3_ (0.05–2 M) or NaOH (0.05–2 M) solutions. During experimental determination, all samples were shaken for 2 h.

In order to study the influence of contact time and temperature on Pt (IV) adsorption process onto the produced adsorbent, all experiments were carried out by mixing 0.1 g XAD7-DB30C10 adsorbent with 25 mL of Pt (IV) solution containing 25 mg Pt (IV) per L. All samples were shaken for different contact times (15, 30, 60, 120, 180 and 240 min) and at different temperatures (298, 308 and 318 K).

Equilibrium studies were performed by using Pt (IV) solutions with different initial concentrations: 15, 25, 45, 75, 100, 150 and 175 mg L^−1^. In order to determine Pt (IV) residual concentration, after the desired contact time has passed, all solutions were filtered, and the Pt (IV) concentration into the residual solutions was measured by atomic adsorption spectrometry.

Adsorption capacity represents the amount of Pt (IV) retained per each gram of adsorbent at a specific time moment, and was evaluated by using the following relation:

(1) $$q=\frac{\left(C_{0}-C_{t}\right)\;V}{m}\;\;\;\;\;\;[\mathrm{mg}\;\mathrm{Pt}\;(\mathrm{IV})/g\;\mathrm{XAD}7\text{-}\mathrm{DB}30C10]$$

where C_o_ and C_t_ are the initial and residual concentrations of Pt (IV) in the solution at moments 0 and t (expressed as mg/L), V is the volume of the solution (L) and m is the mass of the used adsorbent material (g).

In order to recover metallic platinum particles, exhausted material has undergone a thermal treatment carried out in air at 873 K, using a heat rate of 5 K min^−1^.

### 2.4. Factorial Design

The factorial design and the Surface Response experiments were carried out using MINITAB 17.1.0 Statistical Software. Factorial design experiments are used to determine the control factors for studied adsorptive process. These factors present a significant influence on the adsorption process, and based on that was performed a first optimization [29]. The Surface Response experiment only used significant control factors, establishing the non-linear behavior of the adsorption capacity, and made optimization of the adsorption process better, with the purpose to maximize it. Also, possible interactions between control factors have been determined, because such interactions could mask their main effect.

Input variables in this experiment are temperature, time and initial concentration of platinum, and the output one is the adsorption capacity. Numerical values of input variables are: temperature—298 to 318 K, time—15 to 240 min, and Pt (IV) initial concentration—15 to 175 mg L^−1^.

## 3. Results and Discussions

### 3.1. Evaluation of the Interaction between XAD7 Resin and Dibenzo-30-crown-10 Ether

In order to prove functionalization of Amberlite XAD7 with DB30C10 ether, produced adsorbent material was analyzed by using EDX (energy dispersive X-ray analysis), SEM (scanning electron microscopy), FT-IR (Fourier Transform Infra Red) spectroscopy and BET (Brunauer-Emmett-Teller) surface area analysis.

**Figure 2:**
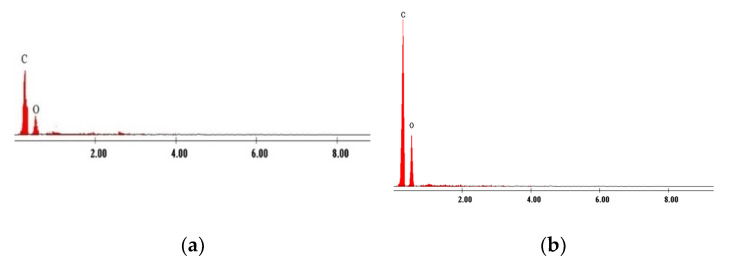
X-ray dispersion (EDX) spectrum of XAD7 before and after DB30C10 immobilized: (**a**) XAD7, (**b**) XAD7-DB30C10.

In Figure 2 are depicted the EDX spectra’s recorded for pure Amberlite XAD7 and for produced adsorbent material obtained after functionalization of Amberlite XAD7 resin with DB30C10 ether.

From spectra’s depicted in Figure 2 can observe that after functionalization of Amberlite XAD7 with DB30C10 crown ether, the heights of carbon and oxygen peaks increases. These increases of the peaks represent a confirmation of the successful functionalization of solid support with used crown ether.

**Figure 3:**
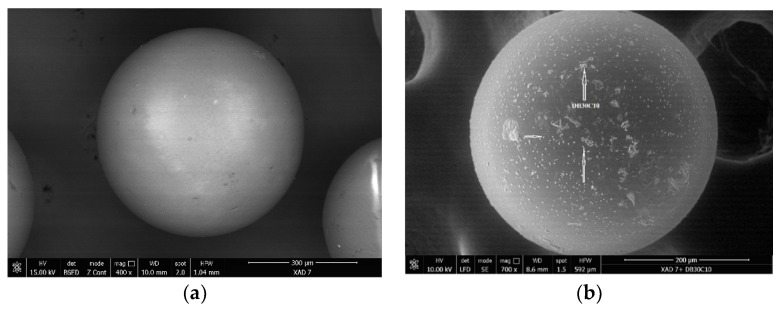
Scanning Electron Microscopy (SEM) of (**a**) Amberlite XAD7, (**b**) Amberlite XAD7-DB30C10.

In next stage were recorded the SEM pictures for pure Amberlite XAD7 resin and for functionalized one, micrographs depicted in Figure 3.

By analyzing the SEM picture presented in Figure 3, it can be observed that at the surface of the XAD7 granules occur some changes, which are affecting the morphology of resin. Presence of this micro-granules fixed onto the Amberlite XAD7 surface is a consequence of the functionalization with DB30C10 crown ether. In this way was confirmed once again the successful functionalization of XAD7 resin with desired crown ether.

**Figure 4:**
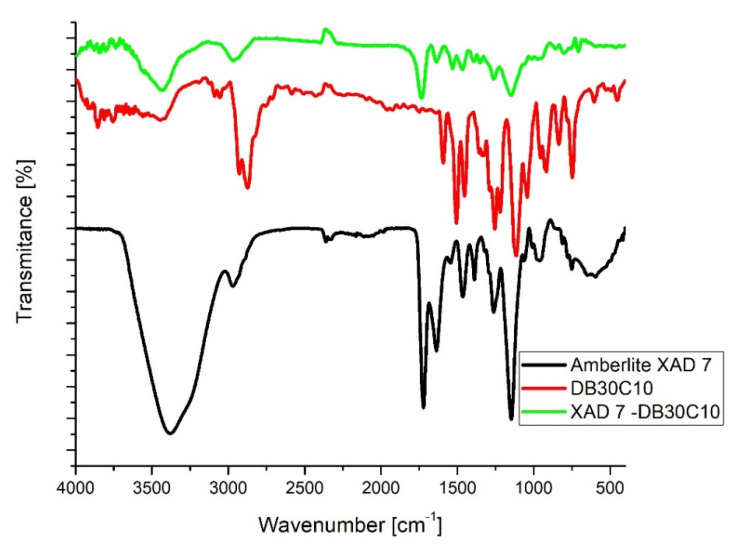
FTIR spectra of XAD7-DB30C10.

Further, the functionalization of Amberlite XAD7 with crown ether was proved by recording the FT-IR spectra’s of pure compounds and of functionalized Amberlite XAD7 (spectra’s presented in Figure 4).

Analyzing the FT-IR spectra depicted in Figure 4 can observe the presence of the bands specific for Amberlite XAD7 resin and for DB30C10 ether [30,31]. Band located at 2300 cm^−1^ it is associated with the stretching vibrations of the C-H bonds from the CH_3_ groups from Amberlite XAD7. Presence of two bands located at 1720 and 1600 cm^−1^ is associated with the existence of C=O and C-C bonds (from Amberlite XAD7) stretching vibrations. Presence of the crown ether onto the studied adsorbent material is proved through the existence of the bands located in the region 1550 and 1000 cm^−1^, bands which can be attributed to the presence of the vibrations of C_aliphatic_-O-C_aromatic_ and respectively C_aliphatic_-O-C_aliphatic_ bonds from crown ether structure.

**Figure 5:**
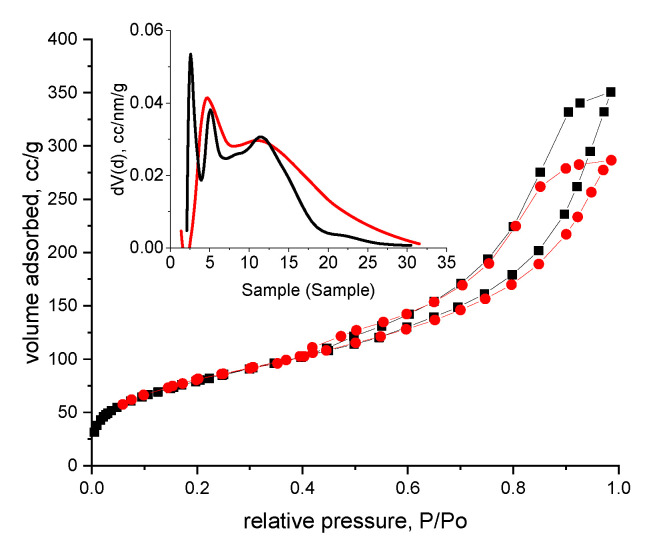
N_2_ adsorption-desorption isotherm with pore size distribution of XAD7 (red line) and XAD7-DB30C10 (black line).

Simultaneously, functionalization of Amberlite XAD7 resin with DB30C10 ether was confirmed by BET analysis (recorded adsorption—desorption isotherms are presented in Figure 5).

Adsorption isotherms presented in Figure 5 were obtained at 77 K after the analyzed sample was degassed at room temperature for 4 h. From the slope of the linear region of the recorded isotherm was calculated the material surface area by using Brunauer, Emmett, Teller equation. Pore size distribution was evaluated by DFT method and is presented as inset figure [29]. Based on the curve depicted in Figure 5 can assume that the adsorption isotherm is the type IV with an H2b hysteresis, accordingly to IUPAC. This means that the shape of the sample pores are similar to ink bottles, but with larger necks, which can be observed from pore size distribution (for analyzed sample the pore size distribution is between 2 and 16 mm). Specific surface area of the sample has a value of 288 m^2^ g^−1^ with a total pore volume of 0.54 cm^3^ g^−1^. Obtained values of these textural parameters could indicate a good adsorption capacity.

### 3.2. Adsorption of Pt (IV) on XAD7-DB30C10

#### 3.2.1. Influence of pH

**Figure 6:**
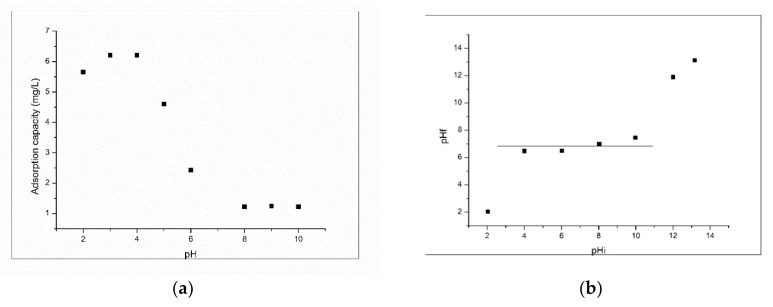
(**a**) Influence of pH on the adsorption capacity of Pt (IV) on XAD7-DB30C10; C_o_ = 25 mg/L; 2 h contact time; 298 K; (**b**) Zero point charge of the adsorbent.

In order to determine the optimum pH range for adsorption of Pt (IV) ions onto the produced adsorbent material, the pH influence over the adsorption capacity of the adsorbent material was studied. Obtained experimental data are presented in Figure 6.

From data depicted in Figure 6a it was observed that an abrupt decrease of the adsorption capacity when the solution pH increases from 5 to 8; also, it was observed that any further increase of pH had no influence on adsorption capacity, which remained constant. Based on obtained data, we can conclude that the adsorption of Pt (IV) onto XAD7-DB30C10 adsorbent occurs with very good results when the solution pH has a maximum value of 4, so all further studies were carried out at pH 4 [32]. From data presented in Figure 6b can observe that the produced adsorbent material present buffering capacities when the solution initial pH is located between 4 and 9, meaning that the material have a pZc between 4 and 9. This means that the XAD7-DB30C10 adsorbent material can be used for adsorptive processes when the system pH is located between 4 and 9.

#### 3.2.2. Influence of Contact Time and Temperature

**Figure 7:**
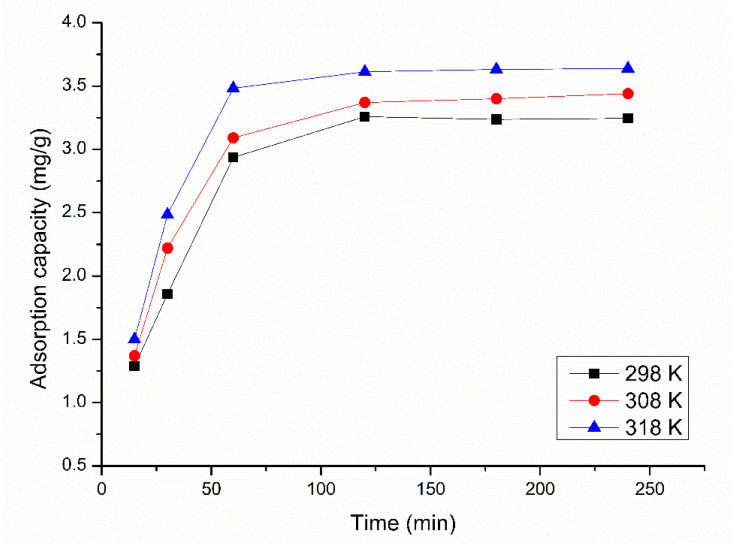
Influence of contact time and temperature on the adsorption capacity of Pt (IV) on. XAD7-DB30C10; Co = 25 mg/L; pH = 4.0.

Experimental data regarding the influence of contact time and temperature on the adsorption capacity of Pt(IV) on XAD7-DB30C10 are presented in Figure 7.

Analyzing the data presented in Figure 7, it was observed that when the contact time is increased up to 60 min, the adsorption capacity has an abrupt increase; after that, any increase of the contact time up to 120 min leads to a slow increase of the adsorption capacity. After 120 min, any further increase of the contact time has no influence on the maximum adsorption capacity, which remains constant. Based on obtained experimental data, we can consider that after 120 min we reached the adsorption equilibrium. So, for any further equilibrium studies the contact time was 120 min [33].

#### 3.2.3. Kinetics and Thermodynamics Studies

Kinetics information regarding Pt (IV) adsorption on XAD7-DB30 C10 were obtained by modeling obtained experimental data obtained for Pt (IV) adsorption from a solution with initial concentration of 25 mg L^−1^ and pH 4 with Lagergren pseudo-first-order model and Ho and McKay pseudo-second-order model.

Integrated form of the pseudo-first-order kinetic model is expressed by Equation (2) [1,2,4].

ln(q_e_ − q_t_) = lnq_e_ − k_1_ t (2)

where q_t_ and q_e_ represent the adsorption capacities at time t and at equilibrium time (120 min), respectively (mg g^−1^) and k_1_ is the specific adsorption rate constant (min^−1^).

**Figure 8:**
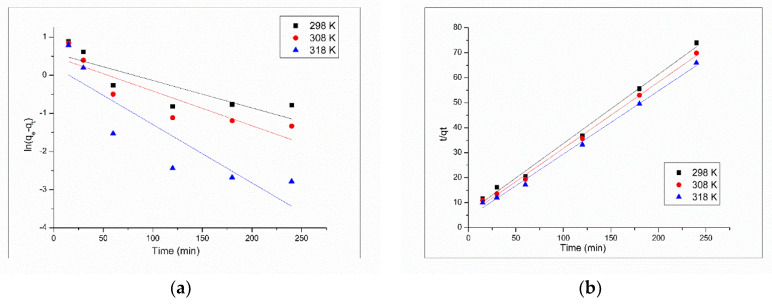
Kinetic models for Pt (IV) adsorption onto XAD7-DB30C10, at different temperatures: (**a**) pseudo-first-order model, (**b**) pseudo-second-order model.

From linear dependence of ln(q_e_ − q_t_) versus time (graph presented in Figure 8a) were evaluated the values of adsorption rate constant (k_1_) and maximum adsorption capacity (q_e_), associated with the pseudo-first-order model.

Linear form of the pseudo-second-order rate expression is given by Equation (3) [1,2,4]:

(3) $$\frac{t}{q_{t}}=\frac{1}{k_{2}q_{e}^{2}}+\frac{t}{q_{e}}$$

where k_2_ is the pseudo-second-order constant (g mg^−1^·min^−1^).

Linear dependence of t/q_t_ versus t, which represent the linear form of pseudo-second-order model is depicted in Figure 8b. Values of pseudo-second-order rate constant (k_2_) and equilibrium adsorption capacity (q_e_) associated with pseudo-second-order model are obtained from the intercept and from the slope of the linear dependence depicted in Figure 8b.

Also, for both used kinetic models were evaluated the values of the regression coefficients (R^2^). All obtained kinetics parameters are summarized in Table 1.

Based on the data presented in Table 1, we can observe that the correlation coefficient obtained when the experimental data were modeled with pseudo-first-order model have a lower value than the value obtained when data were modeled using pseudo-second-order model. Also, when the experimental data were modeled using the pseudo-first-order model, can be observed a huge difference between the calculated maximum adsorption capacity and the experimental determined one. When the experimental data were modeled using the pseudo-second-order model, the theoretically predicted adsorption capacity had a value really close to the experimentally determined one, at all used temperatures. Also, the increase of the adsorption rate constant (k_2_) with the increase of temperature indicate that the adsorption of Pt (IV) on XAD7-DB30C10 is an endothermic process [29].

The correlation coefficient (R^2^) closer to unity indicates that the kinetics of Pt (IV) adsorption on XAD7-DB30C10 is well described by pseudo-second-order kinetic model [32,34].

**Figure 9:**
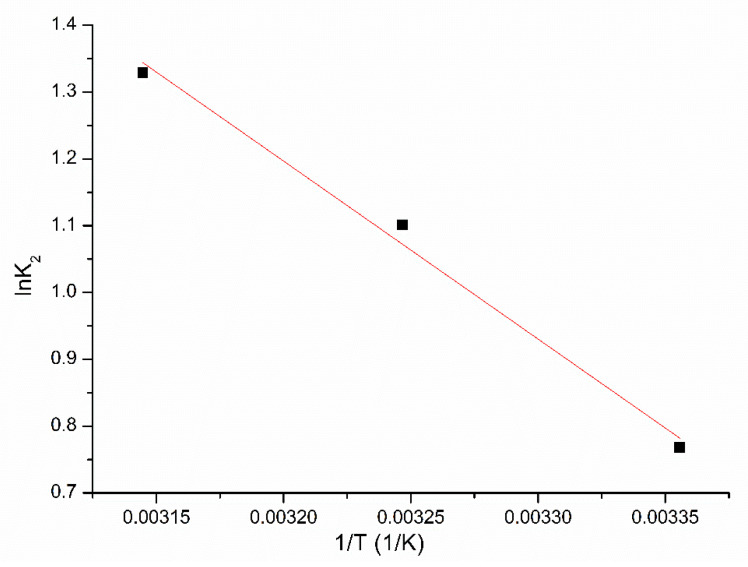
Arrhenius plot of the Pt (IV) adsorption on XAD7-DB30C10.

Further, the value of the activation energy associated with the adsorption process by using the Arrhenius equation was evaluated. Activation energy was determined from linear dependence of lnk_2_ versus 1/T (dependence presented in Figure 9). Speed rate constant was determined by modelling obtained experimental data with pseudo–second–order model.

For studied adsorption process the activation energy have a value of 22.14 kJ mol^−1^. Because the activation energy value obtained for studied adsorption process is bigger than 8 kJ mol^−1^, we can conclude that the Pt (IV) adsorption on XAD7-DB30C10 is a chemical adsorption [35,36,37].

Also, for confirming the studied process is spontaneous, thermodynamic studies were conducted in temperature range 298 to 318 K. Based on obtained experimental data were evaluated the values of thermodynamic parameters: free Gibbs energy (ΔG^0^), free enthalpy (ΔH^0^) and free entropy (ΔS^0^) were calculated by using relations [37,38]:

ΔG^0^ = −RTlnK_d_ (4)

where:

(5) $$K_{d}=\frac{C_{\mathrm{Ae}}}{C_{e}}$$

and

(6) $$\log\;K_{d}=\frac{\Delta S^{0}}{2.3\;R}-\frac{\Delta H^{0}}{2.303\;\mathrm{RT}}$$

where: R is the gas constant, K_d_ is the equilibrium constant, T is the temperature (K), C_Ae_ is the equilibrium concentration Pt (IV) on adsorbent (mg L^−1^), and C_e_ is the equilibrium concentration of Pt (IV) in the solution (mg L^−1^).

Free Gibbs energy, enthalpy and entropy changes represent critical design variables used to estimate material adsorptive performance and to predict the adsorption mechanism. These parameters are the basic requirements for characterization and optimization of adsorptive processes.

**Figure 10:**
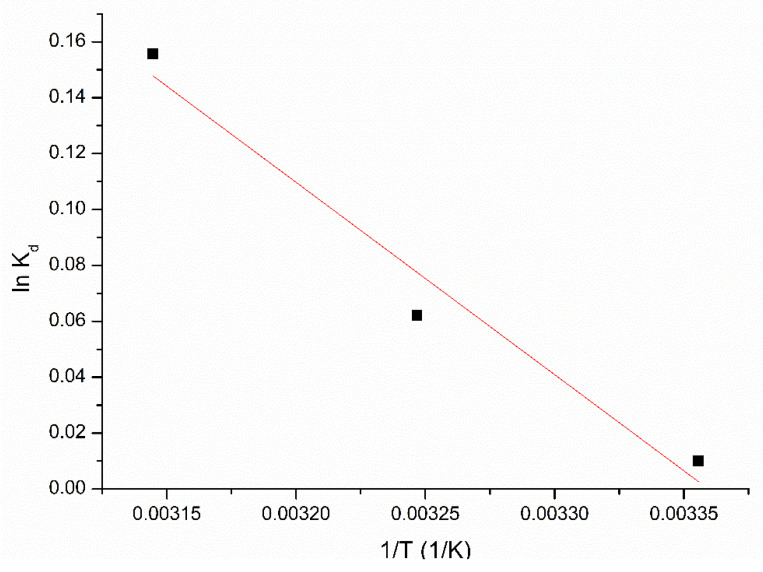
Plot of lnK_d_ vs. 1/T.

Changes of enthalpy and entropy associated with the studied adsorption process are evaluated from the slope and from the intercept of linear dependence of lnK_d_ vs. 1/T (Figure 10). Based on these values can calculate the value of free Gibbs energy. Values of thermodynamic parameters obtained for Pt (IV) adsorption on XAD7-DB30C10 are presented in Table 2.

From the data presented in Table 2 can observe that the ΔH^0^ has a positive value, suggesting that the adsorption process is an endothermic one, so any increase of temperature has a beneficial effect onto the Pt (IV) adsorption. Negative value of free Gibbs energy (ΔG^0^) suggests that the Pt (IV) adsorption on XAD7-DB30C10 is spontaneous and favourable process. Also, can observe that the increase of temperature leads at more negative values of free Gibbs energy meaning that Pt(IV) adsorption speed increases when temperature rises. Positive value of ΔSº suggests that the adsorption speed increase at adsorbent material/solution interface, and the degree of particles clutter increases when the temperature increases [32,33].

#### 3.2.4. Effect of Initial Concentration and Equilibrium Study

**Figure 11:**
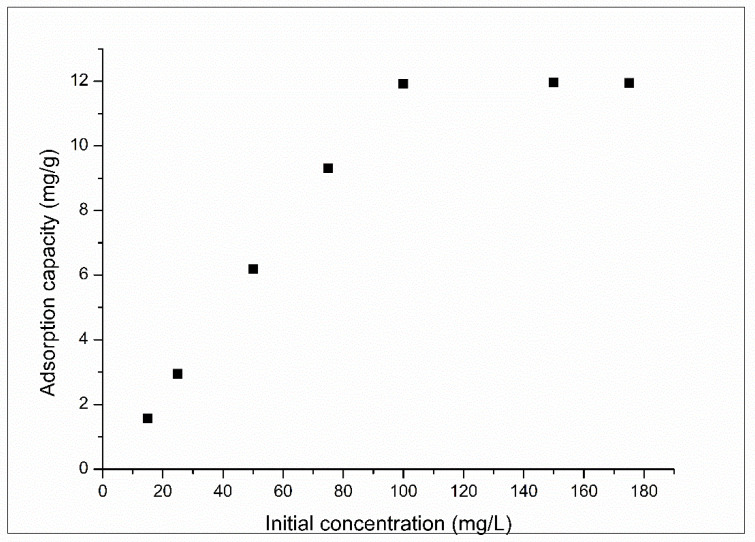
Effect of initial concentration of Pt (IV) on the adsorption process. *C_o_* = (15–175 mg/L); contact time = 120 min; temperature = (273 ± 1) K; pH = 4.

Also was studied the effect of initial concentration on the adsorption process, obtained data are presented in Figure 11.

Based on data presented in Figure 11 can observe that the adsorption capacity increases when the initial concentration of Pt (IV) increases, until a maximum adsorption capacity is reached. This adsorption capacity represents the experimentally determined maximum adsorption capacity of XAD7-DB30C10, having a value of 12.3 mg g^−1^. When adsorption was performed using unfunctionalized material, was obtained a maximum adsorption capacity of 0.03 mg of Pt (IV) per g of used adsorbent material. Maximum adsorption capacity represents another important parameter for designing of adsorptive systems.

Adsorption mechanism can be established by modeling obtained experimental data with different adsorption isotherms. In order to evaluate the adsorption mechanism and to determine the maximum adsorption capacity of Pt (IV) ions onto XAD7-DB30C10 experimental data were modeled using Freundlich, Langmuir and Sips isotherms [35,36,39,40,41].

Linear form of the Freundlich isotherm is expressed by Equation (7) [39]:

(7) $$lnq_{e}=lnK_{F}+\frac{1}{n}lnC_{e}$$

and of the Langmuir isotherm as the following equation:

(8) $$\frac{C_{e}}{q_{e}}=\frac{1}{K_{L}q_{m}}+\frac{C_{e}}{q_{m}}$$

Sips isotherm represent a combination between Freundlich and Langmuir isotherms, which at limits can describe the Langmuir or Freundlich isotherms (Equation (9)) [41]:

(9) $$q_{e}=\frac{q_{s}\;K_{S}\;C_{e}^{1/n_{S}}}{1+K_{S}\;C_{e}^{1/n_{S}}}$$

where: q_e_ is the amount of platinum adsorbed per gram of sorbent at equilibrium (mg g^−1^); C_e_ is the equilibrium concentration of platinum (mg L^−1^); K_F_ and 1/n are characteristic constants that can be related to the relative adsorption capacity of the adsorbent and the intensity of adsorption; q_m_ (maximum adsorption capacity) (mg g^−1^) and K_L_ is a constant related to the free energy of adsorption; q_S_ is the maximum absorption capacity (mg g^−1^); K_S_ is the constant related to the adsorption capacity of the adsorbent and n_S_ is the heterogeneity factor.

**Figure 12:**
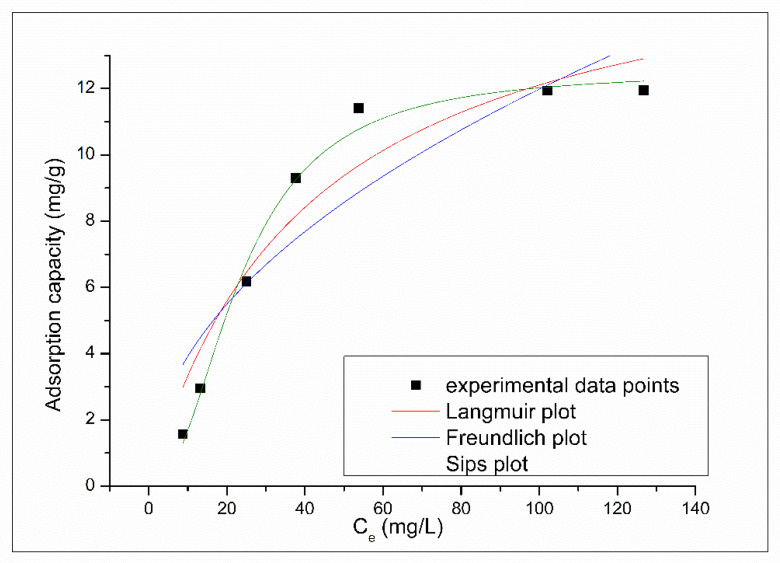
Adsorption isotherm of Pt (IV) onto XAD7-DB30C10.

Results obtained when experimental data were modeled using Freundlich, Langmuir and Sips adsorption isotherms are presented in Figure 12. Based on Freundlich, Langmuir and Sips isotherms presented in Figure 12 were determined the parameters associated with Pt (IV) adsorption, parameters depicted in Table 3.

Analyzing the data presented in Table 3, we can observe that the correlation coefficients obtained for Freundlich and Langmuir isotherms have a lower values compared with the value obtained when experimental data are modeled using Sips isotherm. Lower value of correlation coefficients suggests a restriction in use of Langmuir and Freundlich isotherms to describe the studied adsorption process.

Correlation coefficient of 0.9884 suggests that the Pt (IV) adsorption on XAD7-DB30C10 is better described by the Sips isotherm, which follows the adsorption process for entire concentration range. The value of the maximum adsorption capacity calculated from Sips isotherm have value of 12.53 mg g^−1^, which is very close to the experimental obtained value of 12.3 mg g^−1^. Closer value of maximum adsorption capacity represents a strong confirmation that Sips isotherm describes the adsorption process of Pt (IV) ions onto the studied adsorbent material. Because the calculated value for the coefficient ns is higher than 1 can conclude that the studied adsorption process is a heterogeneous one [29].

To see if the new obtained adsorbent material can be used in real application was compared the maximum adsorption capacity with the one obtained for different adsorbents (data are presented in Table 4).

#### 3.2.5. Platinum Recovery from Exhausted Material

**Figure 13:**
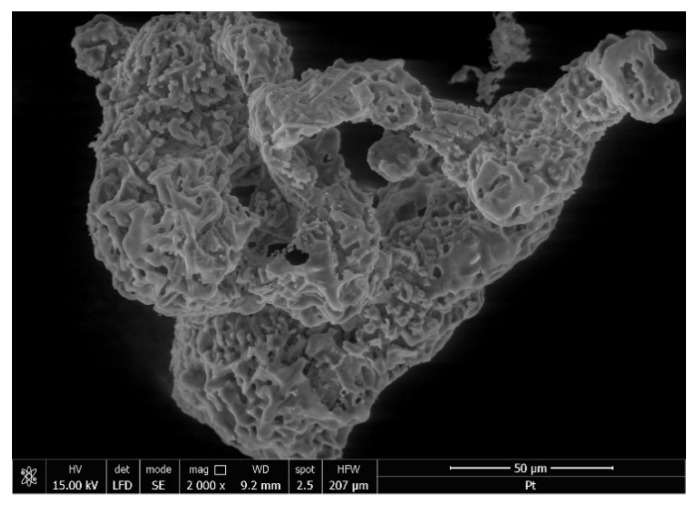
Scanning Electron Microscopy (SEM) of exhausted material with Pt (IV).

**Figure 14:**
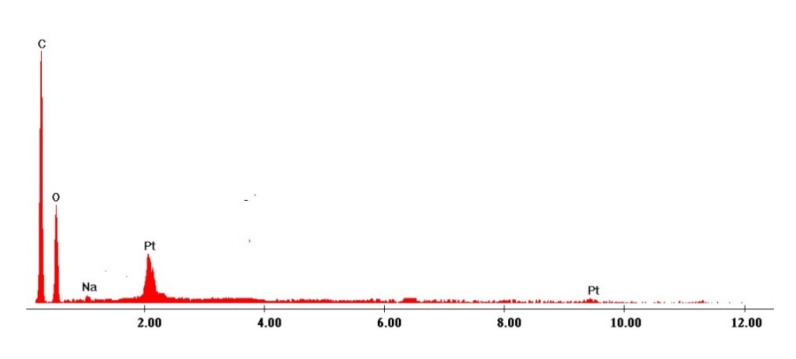
EDX spectrum of exhausted material with Pt (IV).

Metallic platinum was recovered from exhausted adsorbent material by thermal treatment in air at 873 K for 240 min, using a heating rate of 5 K min^−1^, such treatment being necessary to remove the organic part through calcinations. Sample obtained after thermal decomposition was analyzed by scanning electron microscopy—SEM (recorded micrograph is presented in Figure 13) and through X-ray dispersion – EDX (spectra depicted in Figure 14).

By analyzing the SEM micrograph can be obtained information regarding particle morphology and distribution of platinum particles in adsorbent ash. From the EDX spectra can observe the presence of Pt (IV) specific peaks, being a confirmation of Pt (IV) adsorption onto the produced adsorbent material. Based on recorded EDX spectra was obtained the chemical composition of calcinated sample, composition presented in Table 5.

**Figure 15:**
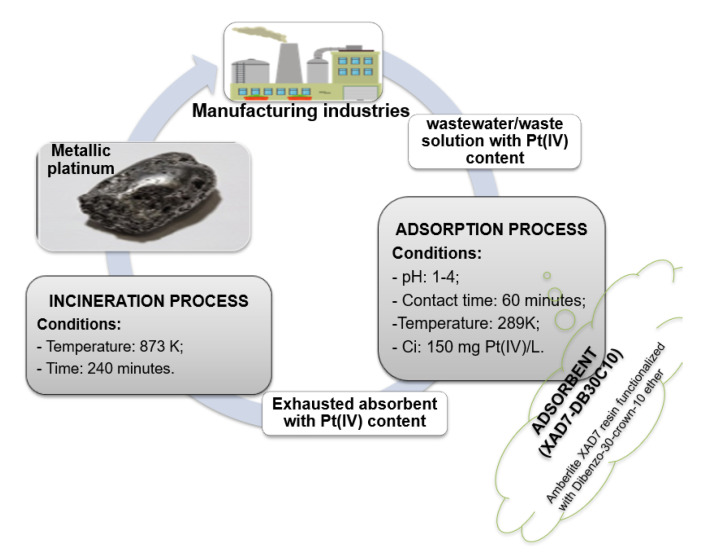
Proposed process for recovery metallic platinum from wastewater/waste solution.

Based on obtained experimental data, we propose a process for direct recovery of metallic platinum from wastewater/waste solutions through adsorption on XAD7-DB30C10, followed by thermal decomposition of exhausted adsorbent (Figure 15).

#### 3.2.6. Factorial Design

##### Linear Experiments

**Figure 16:**
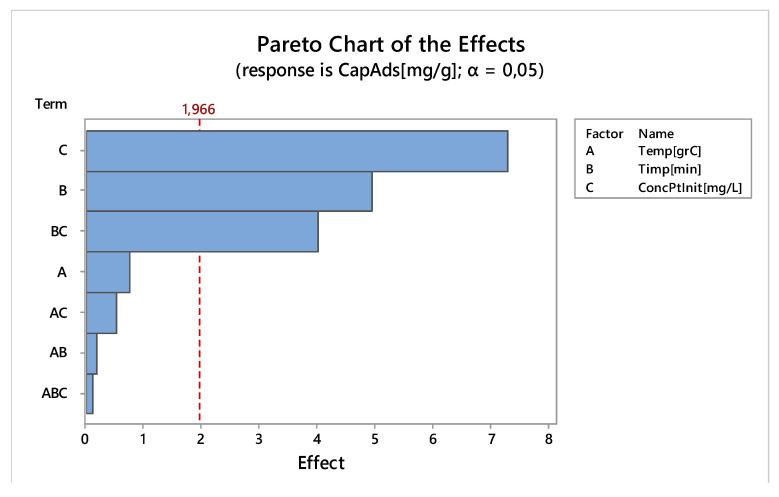
The effect of control parameters on the adsorption capacity.

Main objective of linear design experiments was to establish control factors (contact time, temperature and initial concentration) with significant effect on adsorption capacity (q, mg g^−1^), to establish connections between control factors and making a first optimization of the process. The factors of control of the factorial design were randomly distributed through sets of experiments. Significant effect control factors can be seen in the diagram Pareto (Figure 16).

**Figure 17:**
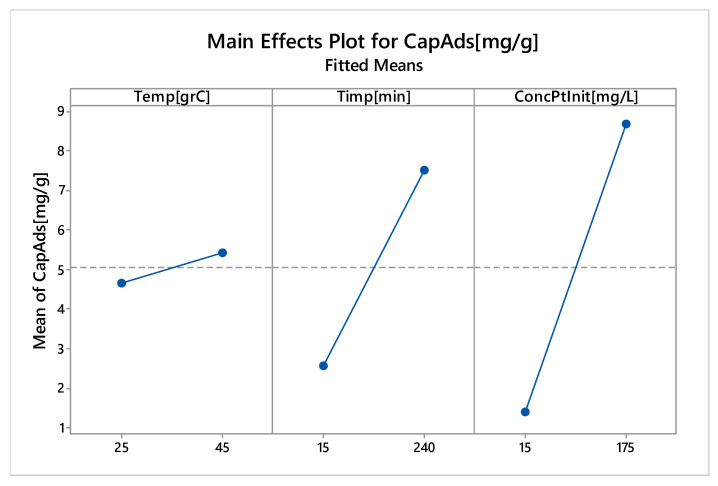
Main effects of control factors on adsorption capacity.

Pareto diagram show that a significant effect on the adsorption capacity have: initial concentration of platinum and the contact time. The main effects of the control factors on the adsorption capacity are shown in Figure 17.

**Figure 18:**
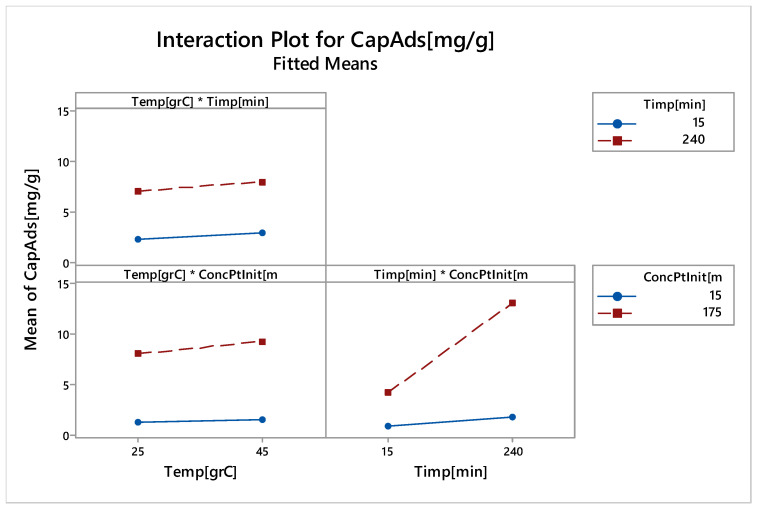
Interactions between control factors with responses of adsorption process (capacity adsorption).

It should be noted that all the main effects are positive. It can also be observed that the significant effects, with the highest slope, have the contact time and the initial platinum concentration (also evidenced by Pareto diagram). The data obtained by modelling the adsorption process are in close correlation with the results obtained from the laboratory adsorption studies. Interactions between the control factors with the adsorption process response (adsorption capacity) are shown in Figure 18.

Interactions occur when the lines in Figure 18 are not parallel. Thus, a strong deviation from parallelism highlights a strong interaction between control factors. In Figure 18, there is a significant interaction between the initial platinum concentration and time (see also Pareto diagram).

**Figure 19:**
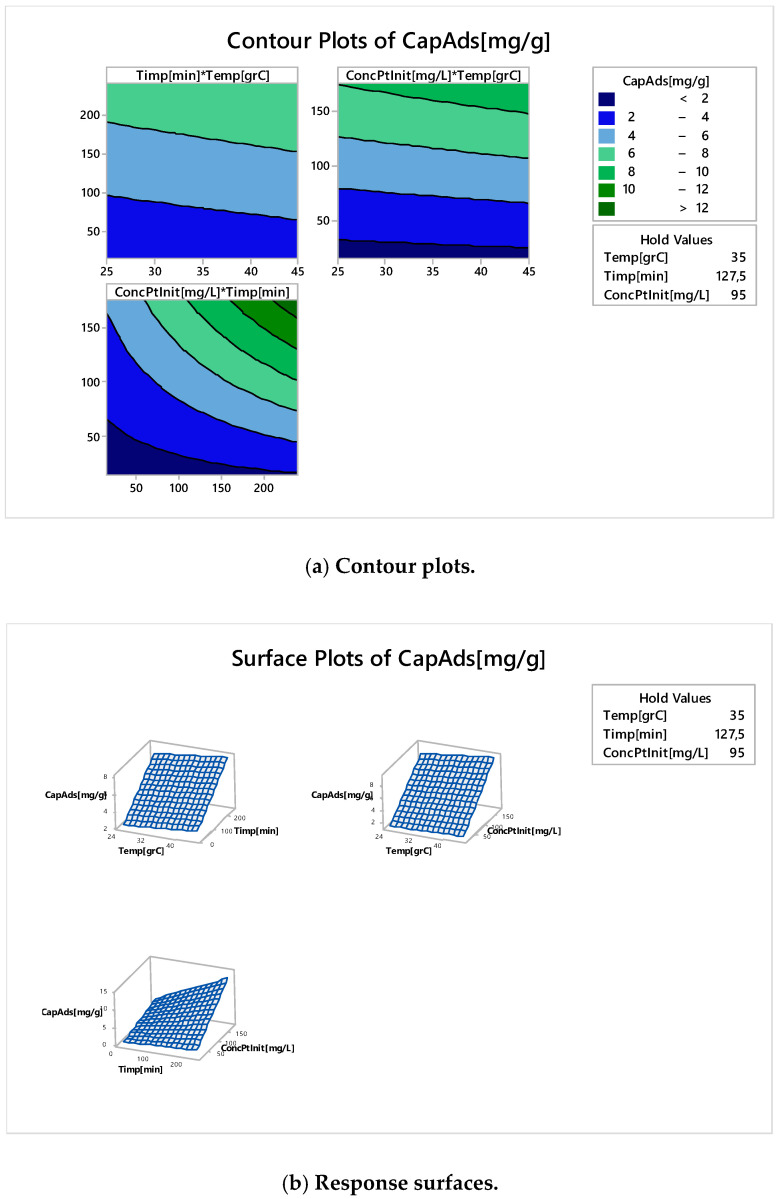
Contour Plots (**a**) and response surfaces (**b**).

In the Figure 19 are shown the contour plots (Figure 19a) and responses surfaces (Figure 19b) for all studied control factors (contact time of the initial concentration and temperature) if the output response is the adsorption capacity.

It can be noticed that the level curves (Figure 19a) do not indicate non-linearity, except for the graph ConcPtInit* Time, which shows a slight non-linearity. From the response surface curves (Figure 19b) we can see that the first two graphs are linear and the third one has slight non-linearity (see Contour Plots).

**Figure 20:**
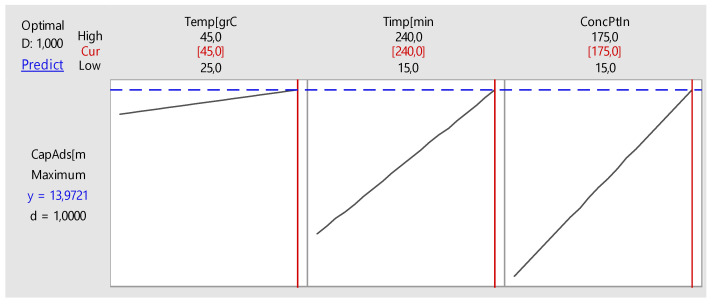
Global optimization process platinum adsorption.

A global optimization of the chemical process was made, in our case platinum adsorption. It establishes the best setting of control factors (contact time, temperature and initial concentration) for maximum adsorption capacity (q). The result of global optimization is shown in Figure 20.

In adsorption process optimization maximum adsorption capacity is achieved at a maximum range of factors variation. By higher values for temperature, time and initial concentration, the adsorption capacity can be increased (linear). Growth may be limited for objective reasons of limiting the values of control factors.

##### Nonlinear Experiments—Response Surface Design (RSD). Optimization of the Adsorption Process

**Figure 21:**
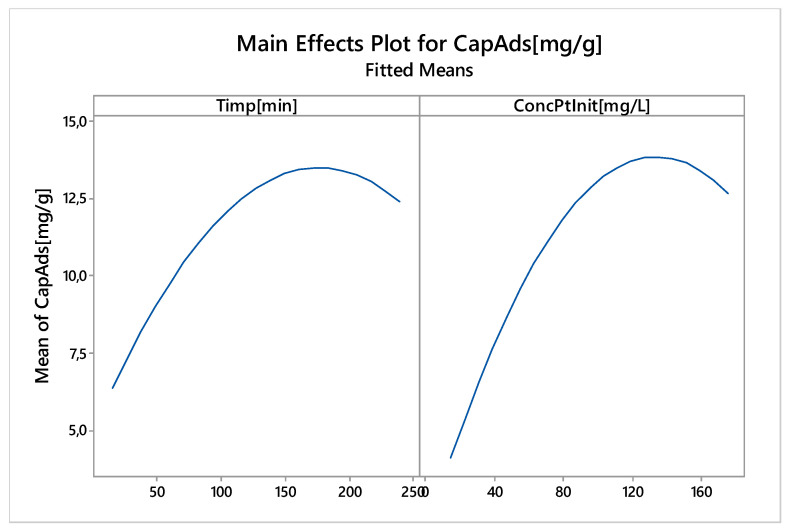
Main effects of controlled factors on adsorption capacity.

The main objective of the nonlinear experimental technique, RSD, was the nonlinear optimization of one of the chemical process responses, in our case the adsorption: adsorption capacity (q, mg g^−1^) depending on the input variables that have a significant effect on it. These input variables are the contact time and the initial platinum concentration, which was determined in linear experiments. In the Central Composite Design, the factors were randomly distributed through experiment sets. The main effects of controlled factors are shown in Figure 21.

It is noted that for both input variables (contact time and initial platinum concentration) there is a non-linear effect, with maximum adsorption capacity being achieved for a contact time around 180 min and an initial concentration around 135 mg L^−1^.

**Figure 22:**
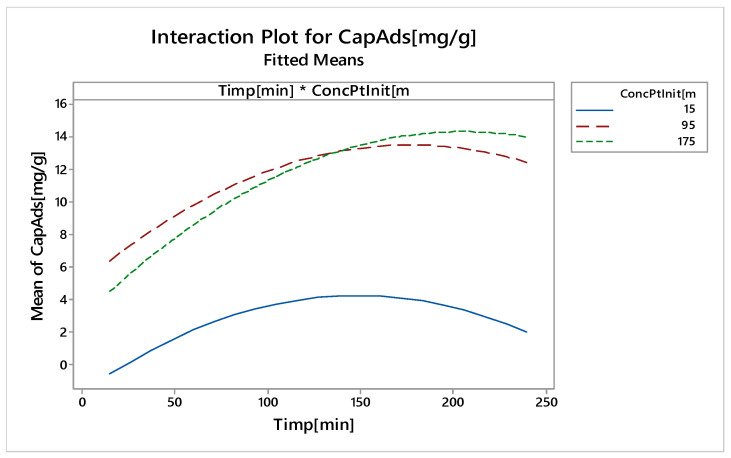
Interactions between input variables.

The interactions that occur between the input variables are shown in Figure 22.

Curves for the initial platinum concentration of 15 and 95 mg L^−1^ are parallel, which means there are no interactions. It can be observed that there were slight interactions between the initial concentration of 95 mg L^−1^ and 175 mg L^−1^. Practical interactions do not mask the main effects.

**Figure 23:**
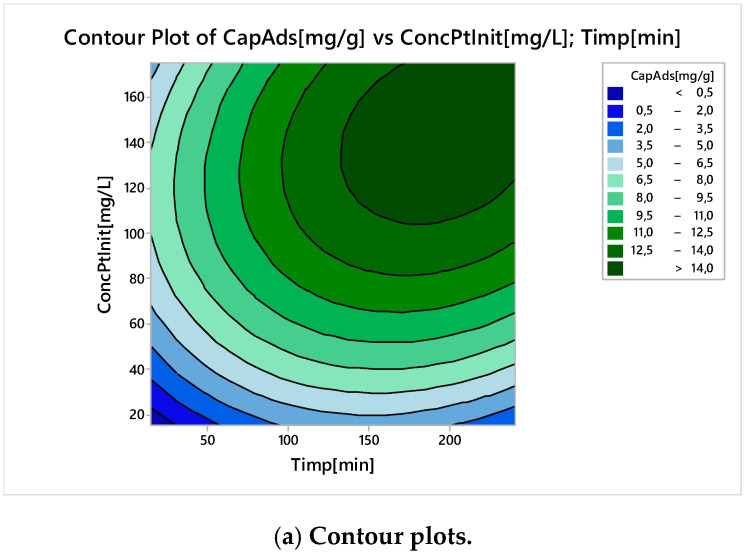

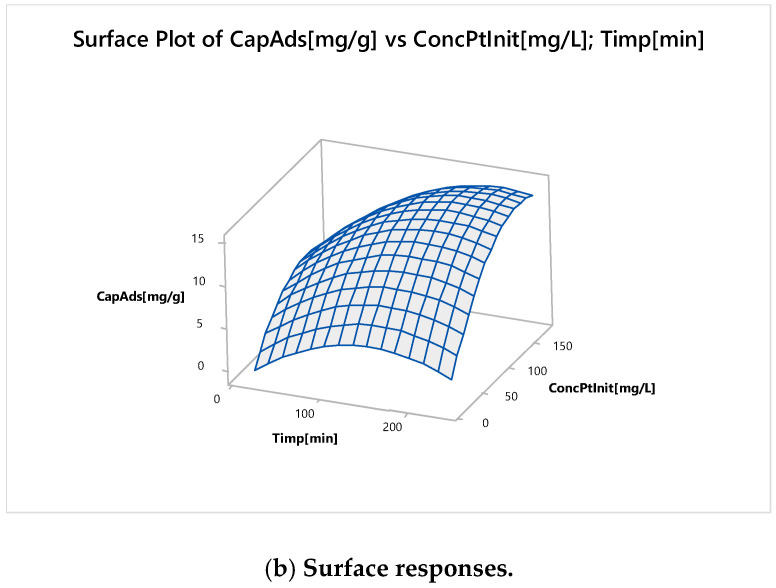
Contour plots (**a**) and surface responses (**b**) for time and initial platinum concentration when response is the adsorption capacity.

In Figure 23 are illustrated Contour Plots (Figure 23a) and the Surface Responses (Figure 23b) for two control factors (time and initial concentration).

The adsorption capacity had nonlinear behaviour, with a maximum at a contact time of 190 min, and an initial concentration of around 140 mg L^−1^.

**Figure 24:**
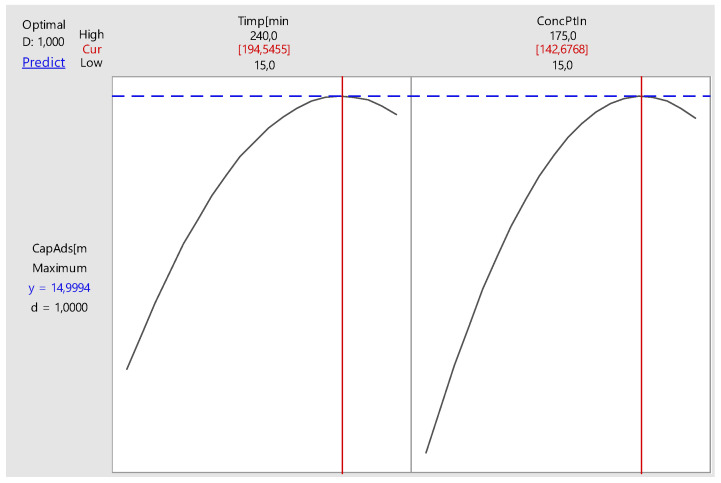
Optimization of the adsorption process.

Using the optimization feature provided by the software MINITAB, the optimal time setting and initial concentration can be determined for maximum adsorption capacity. This optimization of the response of adsorption process is presented in Table 6 and Figure 24.

By optimizing the adsorption process, a maximum adsorption capacity of 15.03 mg g^−1^ was achieved at a 194.5 min and an initial concentration of 142.67 mg L^−1^ with a confidence interval of 95%.

## 4. Conclusions

In the present paper we described the preparation of new adsorbent material through functionalization of Amberlite XAD7 resin with dibenzo-30-crown-10 ether (DB30C10). This material has been obtained using solvent impregnated resin method (SIR method), when nitrobenzene was used as solvent during the dry impregnation of Amberlite XAD7 with DB30C10. Functionalization of polymeric support has been demonstrated by FT-IR spectroscopy, SEM, EDX and BET analysis.

Adsorption experiments were carried out in order to understand how the adsorption process is influenced by various parameters, such as: Pt(IV) initial concentration, contact time, pH and temperature. Based on these experiments was possible to find the optimum conditions for the Pt (IV) recovery on XAD7- DB30C10.

Obtained experimental data proved that the maximum adsorption capacity was obtained when the process was driven at pH 4. Another important parameter for adsorptive processes is represented by the contact time; based on obtained experimental data was established that the adsorption equilibrium was reached after 120 min.

Information regarding the adsorption kinetic were obtained by modelling obtained experimental data with pseudo-first-order and pseudo-second-order models. Data are better described by the pseudo-second-order model, Pt (IV) adsorption being a chemical adsorption process. The thermodynamic parameters evaluated from the Van’t Hoff equation indicate that the adsorption process is spontaneous and exothermic. Further adsorption data were modeled using Langmuir, Freundlich and Sips adsorption isotherms, proving that the Pt (IV) adsorption on XAD7-DB30C10 is better described by Sips model.

Amberlite XAD7-DB30C10 adsorbent material present a maximum adsorption capacity of 12.5 mg Pt (IV) per g, representing a possible candidate for Pt(IV) ions recovery from residual solutions. After adsorption metallic platinum can be relatively easily recovered by calcination of exhausted adsorbent material in air.

Designing experiments was used to optimize the adsorption process, leading to the optimal values for control factors. Factorial Design and Surface Response Design of experimental data have demonstrated that the platinum absorption process from aqueous solutions depends mainly on contact time and initial concentration and does not depend too much on process temperature. By optimizing the process, a maximum adsorption capacity of 15.03 mg g^−1^ was achieved for a contact time of 190 min, and an initial concentration of 141.06 mg L^−1^ with a confidence interval of 95%.

The experimentally obtained data is confirmed by the data obtained through the factorial design. Thus, the optimal values of parameters determined experimentally are: contact time 120 min, temperature 25 °C, and platinum initial concentration of 150 mg L^−1^. The maximum adsorption capacity obtained experimentally was 12.3 mg g^−1^, while the values obtained by the factorial design experiments were: contact time 190 min, temperature 45 °C and the platinum initial concentration of 141.06 mg L^−1^, resulting an adsorption capacity of 15.03 mg g^−1^. From the design experiments is noticed using a higher temperature leads to a slightly higher adsorption capacity but not being cost efficient will not be industrially pursued.

At the same time, Design of Experiments has helped establish optimal settings for chemical processes, with minimal time and cost.

## Figures and Tables

**Table 1:** Kinetic parameters for the adsorption of Pt (IV) onto XAD7-DB30C10.

Temperature (K)	Pseudo-First-Order Model				Pseudo-Second-Order Model			
	q_e exp_ (mg g^−1^)	k_1_ (min^−1^)	q_e_, Kinetic Plot (mg g^−1^)	R^2^	q_e exp_ (mg g^−1^)	k_2_ (g mg^−1^·min^−1^)	q_e_ Kinetic Plot (mg g^−1^)	R^2^
298	3.26	0.0072	1.79	0.7154	3.26	2.15	3.62	0.994
308	3.37	0.0091	1.64	0.7930	3.37	3.00	3.95	0.9967
318	3.61	0.0153	1.27	0.7787	3.61	3.77	3.95	0.9955

**Table 2:** Thermodynamic parameters for the adsorption of Pt (IV) on XAD7-DB30C10.

ΔH^0^ (kJ/mol^−1^)	ΔS^0^ (J/mol∙K)	ΔG^0^ (kJ/mol)			R^2^
		298 K	308 K	318 K	
5.72	19.21	−6.4	−6.9	−7.3	0.9676

**Table 3:** Parameters of isotherm model for the adsorption of Pt (IV) on XAD7-DB30C10.

**Langmuir Isotherm**			
**q_m,exp_ (mg g^−1^)**	**K_L_ (L mg^−1^)**	**q_L_ (mg g^−1^)**	**R^2^**
12.3	0.024	17.1	0.9085
**Freundlich Isotherm**			
**K** **_F_** **(mg g^−1^)**	**1/n_F_**		**R** **^2^**
1.28	0.484		0.8026
**Sips Isotherm**			
**K** **_S_**	**q** **_S_** **(mg g^−1^)**	**1/n_S_**	**R** **^2^**
1.0 × 10^−2^	12.5	1.2	0.9884

**Table 4:** Comparison to other materials for Pt (IV) recovery.

**Adsorbent**	**Adsorption Conditions**	**Adsorption Capacity [mg/g]**	**References**
Fungus aspergillus sp. immobilized on Cellex-T	pH = 1; 298 K	0.47	[42]
Cross-linked carboxy methyl chitosan hydrogels	pH = 3.3; contact time 120 min; 298 K	1.16	[43]
Functionalized acrylic copolymers	pH = 1; time = 60 min, 303 K,	1.10	[44]
DMA persimmon waste gel (DMA-PW)	pH = 0.9; contact time = 24 h, 343 K,	1.28	[45]
Amberlite XAD-7- dibenzo-30-crown-10 ether	pH = 4; contact time = 120 min; 293 K	12.3	Present paper

Analyzing the data depicted in previous table can observe that the ne produced adsorbent (XAD7-DB30C10) present a very good efficiency for Pt (IV) recovery from waste solution through adsorption, representing a real alternative for classical adsorbents.

**Table 5:** Chemical composition.

Elem	Wt. %
C	45.18
O	39.2
Na	6.39
Pt	9.23
Total	100

**Table 6:** Optimization the response of the platinum adsorption process.

Parameter	Minimum	Target
Adsorption capacity, mg g^−1^	10	14
**Global Solution**		
Time, minutes	195	
Initial Concentration, mg L^−1^	143	
**Predicted Response**		
Adsorption Capacity, mg g^−1^	15	
